# Role of Immunity and Vaginal Microbiome in Clearance and Persistence of Human Papillomavirus Infection

**DOI:** 10.3389/fcimb.2022.927131

**Published:** 2022-07-07

**Authors:** Lungelo Ntuli, Andile Mtshali, Gugulethu Mzobe, Lenine JP Liebenberg, Sinaye Ngcapu

**Affiliations:** ^1^ Department of Medical Microbiology, School of Laboratory Medicine and Medical Science, University of KwaZulu-Natal, Durban, South Africa; ^2^ Centre for the AIDS Programme of Research in South Africa (CAPRISA), Durban, South Africa

**Keywords:** human papillomavirus, cellular, cytokines, inflammation, vaginal microbiota

## Abstract

Cervical cancer disproportionately affects women of reproductive age, with 80% of cases occurring in low- and middle-income countries. Persistent infection with high-risk human papillomavirus (HPV) genotypes has been described as the most common non-systemic biological risk factor for the development of cervical cancer. The mucosal immune system plays a significant role in controlling HPV infection by acting as the first line of host defense at the mucosal surface. However, the virus can evade host immunity using various mechanisms, including inhibition of the antiviral immune response necessary for HPV clearance. Pro-inflammatory cytokines and the vaginal microbiome coordinate cell-mediated immune responses and play a pivotal role in modulating immunity. Recently, diverse vaginal microbiome (associated with bacterial vaginosis) and genital inflammation have emerged as potential drivers of high-risk HPV positivity and disease severity in women. The potential role of these risk factors on HPV recurrence and persistence remains unclear. This article reviews the role of cellular or cytokine response and vaginal microbiome dysbiosis in the clearance, persistence, and recurrence of HPV infection.

## Introduction

Human Papillomavirus (HPV), a primary cause of genital warts and cervical cancer, is the most common sexually transmitted infection (Burchell et al., 2006). Despite efforts to implement prophylactic HPV vaccination in young women, cervical cancer is the fourth most common cancer among sexually active women globally (Sung et al., 2021). A majority (~90%) of women clear HPV infections spontaneously within 6–18 months (Myers et al., 2000; Plummer et al., 2007), with very few progressions from precancerous lesion to invasive cervical cancer (Stanley, 2008). Several studies have hypothesized that host defense mechanisms, the genital microbiome, and other factors in the female genital tract play a role in the clearance and persistence of HPV, including the risk of developing cervical cancer (Moscicki et al., 2001; Daud et al., 2011; Łaniewski et al., 2018; Liebenberg et al., 2019; Onywera et al., 2019).

Innate and adaptive immune responses represent the first line of host defense at the mucosal surface against pathogens such as HPV (Lieberman et al., 2008, Barros et al., 2018). Several studies have demonstrated a relationship between genital mucosal cytokine concentrations and the control or elimination of HPV infection in the cervix (Liebenberg et al., 2019, 12–Li et al., 2019). It is hypothesized that cytokine response occurs within days after the establishment of an HPV infection and is subsequently reversed when HPV clearance has been effectively achieved by appropriate effector cells (12, O'Byrne and Dalgleish, 2001; Balkwill and Mantovani, 2001; Hawes and Kiviat, 2002; Amaral et al., 2006). The initial response against HPV infection includes mature antigen presenting cells (APCs) that secret cytokines and contribute in activation and recruitment of other immune cells to the site of infection (Stanley, 2006). Upon interaction with mature APCs, naïve CD4+ and CD8+ T cells differentiate into various T helper effector lineages and cytotoxic T lymphocytes (CTLs), respectively, which needed for the effective clearance of HPV (Steele et al., 2002; Stanley, 2006). However, these cells do not entirely prevent disease progression. HPV can use various immune evasion mechanisms to limit anti-viral activity of immune response, resulting in HPV infection tolerance in the host’s immune system. HPV infection could affect the differentiation of monocytes into mature DCs and distinctively affect the functionality of CD4+/CD8+, and regulatory T cells (Song et al., 2015). Increased secretion of anti-inflammatory cytokine IL-10 in Th2 response has been associated with compromised innate and adaptive immune defense and cervical lesion progression during high-risk HPV infection (Peghini et al., 2012; Scott et al., 2013; Lin et al., 2019). Although studies have demonstrated the anti-viral activities of immune cells, less is known about the cell-mediated mucosal immune response to HPV incidence, persistence, and clearance.

Emerging evidence suggests that women with a low relative abundance of vaginal *Lactobacillus* species and high proportions of the *Gardnerella*, *Sneathia*, and *Atopobium* genera are less likely to clear HPV infection (Gillet et al., 2011; Gao et al., 2013; Brotman et al., 2014; Mitra et al., 2015; Audirac-Chalifour et al., 2016; Łaniewski et al., 2018; Norenhag et al., 2020). Two recent meta-analyses summarizing findings from several microscopy or molecular studies have reported that women with *Lactobacillus*-enriched microbiomes were less likely to acquire HPV infection compared to women with overgrowth of bacterial vaginosis (BV)-linked bacteria (*Gardnerella*, *Atopobium*, and *Prevotella*) (Brusselaers et al., 2019; Norenhag et al., 2020). A *Lactobacillus gasseri*-enriched microbiome was associated with rapid rates of HPV clearance (Brotman et al., 2014; Di Paola et al., 2017), while a *L. iners* dominant microbiome was commonly reported in women presenting with cervical intraepithelial neoplasia (CIN). Several studies further reported an association between BV-linked microbial communities (*Gardnerella, Prevotella, Dialister, Streptococcus, Ureaplasma, Megasphaera*, and *Mycoplasma*) and persistent infection with high-risk HPV that may result in the development of CIN (Norenhag et al., 2020; Wei et al., 2020). Furthermore, specific vaginal microbiota may modulate host immune responses, including critical antiviral and anti-tumor immunity components in the female genital tract (Doerflinger et al., 2014; Anahtar et al., 2015). Diverse microbial communities have been closely associated with altered innate immune responses, host susceptibility to infection (Anahtar et al., 2015; Gosmann et al., 2017; Mtshali et al., 2021), and development of cervical diseases, but proven causality remains unclear.

While there is evidence of a relationship between the vaginal microbiome, host immune responses, and HPV infection, a link between the vaginal microbiome, host responses, and the progression to HPV-associated cervical cancer remains unclear. Therefore, understanding the concept of vaginal microbiome fluctuations and associated host immune responses during HPV infection stages could shed light on possible mechanisms associated with cervical carcinogenesis. This review summarizes the role of vaginal microbiome fluctuations, cytokine, and cellular immune response during HPV infection, persistent and clearance.

## HPV Genotypes and Screening

HPV is a member of the non-enveloped double-stranded DNA papillomaviridae family that infects squamous epithelium found beneath the foreskin of the penis, the scrotum, vulva, vagina, cervix, skin, and anus (Doorbar et al., 2015; Harden and Munger, 2017). The papillomavirus structure is icosahedral with approximately 50–60 nm and contains 8000 base pairs (Harden and Munger, 2017; Gupta and Mania-Pramanik, 2019). HPV infection is classified into five major genera, *Alphapapillomavirus, Gammapapillomavirus, Betapapillomavirus, Deltapapillomavirus*, and *Mupapillomavirus*, with only *Alphapapillomavirus, Gammapapillomavirus* and *Betapapillomavirus* affecting humans (Harden and Munger, 2017). Of the genera affecting humans, *Alphapapillomavirus* is the most common genus infecting the genital tract (International Agency for Research on Cancer, 2018).

More than 200 genital HPV genotypes have been molecularly characterized, with some categorized into low-risk genotypes (causing genital warts) while others are categorized as high-risk genotypes (causing CIN and cervical cancer) (Juckett and Hartman-Adams, 2010). High-risk HPV types include HPV 16, 18, 31, 33, 35, 39, 45, 51, 52, 56, 68, and 59, with HPV 16 and 18 being the most prevalent (accounting for 70% of cervical cancer cases) genotypes (Clifford et al., 2017). The most common low-risk HPV include HPV 6 and 11, accounting for ~90% of genital warts and rarely developing into cancer (Ebrahim et al., 2016). Cervical cancer precursor lesion screening uses biopsy, colposcopy and acetic acid test, Pap smear, and nucleic acid-based tests (Agorastos et al., 2010). A Pap smear is the main screening tool to identify precancerous cells in the cervix, and any observed abnormalities are further evaluated with colposcopy, biopsy, and molecular-based tests (Agorastos et al., 2010).

## HPV Treatment and Prevention Strategies

Although there is no effective treatment for the virus itself, oncogenic HPV infections and subsequent HPV-associated lesions can be prevented by vaccination (WHO, 2022). There are three commercially available prophylactic vaccines targeting different HPV types, including the bivalent (Cervarix^®^), quadrivalent (Gardasil^®^), and nonavalent (Gardasil^®^9) vaccines (Arbyn and Xu, 2018). The bivalent vaccine protects against the two commonly known high-risk oncogenic types, HPV 16 and HPV 18 (Juckett and Hartman-Adams, 2010). The quadrivalent vaccine acts against these and two genital wart-causing low-risk types (HPV 6 and 11) (Stern et al., 2000; Hancock et al., 2018). The nonavalent vaccine is directed against 9 HPV genotypes, the four collectively targeted by the bivalent and quadrivalent vaccines (HPV 16, 18, 6, and 11) and five additional strains associated with cervical cancer (HPV 31, 33, 45, 52, 58) (Lekoane et al., 2019). The adverse effects of HPV vaccination are normal mild local reactions and their safety has resulted in effective HPV vaccination program in majority of developed countries, but the uptake of these vaccinations by native populations is low (Poirier et al., 2021). HPV vaccination is provided to adolescents from 9 to 12 years (Wang et al., 2020) to prevent HPV infections, abnormal cervical cytology, and cervical cancer (Palefsky, 2020). However, these primary prevention strategies are unable to protect against all types of HPVs or eradicate the existing HPV-infected cells, as capsid proteins are established either before viral entry, or in the terminally differentiated epithelium (Castle and Maza, 2016). Women identified with precancerous lesions can be treated using cryotherapy or thermal ablation, and invasive cervical cancer can be treated by surgery, chemotherapy, and radiotherapy (WHO, 2022). Collectively, the partial protection prophylactic vaccines has against certain HPV types that are associated with cervical cancer justifies the need for continual screening and development of additional therapeutic options to resolve cases post-infection.

## HPV Infection in Young Women

**Figure 1:**
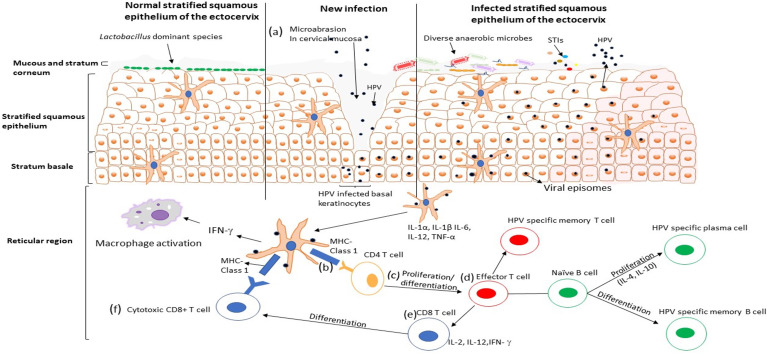
HPV infection cycle in the ectocervix of the female genital tract. **(A)** The HPV virus particles penetrate the stratified squamous epithelium through microabrasion. The virus particles infect the basal keratinocytes in the basement membrane and establish an infection, viral replication occurs, and the infected cells are transported up the epithelium. **(B)** Viral particles are taken up by dendritic cells that secrete T cells attracting pro-inflammatory cytokines and chemokines (IL-1 α, IL-1β, IL-6, TNF-α, IL-12), which may facilitate activation and recruitment of CD4+ T cells. CD4+ T cells recognise HPV antigen presented by the dendritic cells and **(C)** undergoes proliferation and differentiation into the effector T cell. **(D)** The effort T cells [which proliferate into HPV specific memory T cell and **(E, F)**cytotoxic CD8+ T cell] binds with the naïve B cells, which differentiate to HPV specific plasma cells and HPV specific memory Bcells. Activation of cellular and humoral immunity is associated with clearance of HPV infection.

Young women in sub-Saharan Africa continue to bear a disproportionate burden of HPV (Pimple and Mishra, 2022), where the HIV epidemic has reached its greatest scale (UNAIDS, 2017). Adolescent girls and young women are more susceptible to HPV infection than their male counterparts (Bruni et al., 2010; Insinga et al., 2010; Edna Omar et al., 2017). Bruni *et al.* (2010) showed that HPV infection peaks in younger women around the age of sexual debut and declines during later decades of life (Bruni et al., 2010). The unique vulnerability of women to HPV infection comes from several different behavioral and biological factors. While the risk for infection differs from person to person, increased number of sexual partners, early sexual debut, the use of intravaginal insertion products, uncircumcised male partners, and the number of prior pregnancies is some of the documented behavioral risk factors in women (Sasagawa et al., 2012; Dreyer, 2018). Several biological factors, including vaginal surface, the relatively larger area of cervical epithelium undergoing squamous metaplasia, microabrasion in cervical mucosa (**Figure 1A**), immunosuppression, co-occurrence of STIs, disturbance of vaginal microenvironment and menstruation, defective immune responses associated with genetic variations, and condomless sex, may predispose women to become infected with HPV (Insinga et al., 2010; Dempsey and Mendez, 2010). Despite evidence for an etiological role for HPV, these risk factors remain largely unexplored. Understanding the role of behavioral and biological risk factors for HPV infection in adolescent girls and young women could be crucial to developing effective ways to prevent HPV infection, and prophylactic vaccines.

## Immune Response and Evasion Mechanisms

Women are particularly vulnerable to HPV and are predominantly infected through heterosexual transmission (Bosch et al., 2002; Bruni et al., 2010; Branstetter et al., 2017). HPV infections are localized to keratinocytes. The mucosal epithelium provides the first line of defense against pathogen entry and mediates the initial host immune response against HPV (Stanley, 2006). Pathogens like HPV are detected by an intricate matrix of innate and adaptive immune responses of the lower and upper female genital tract.

### Innate Immune Response

Innate immune cells like neutrophils, monocytes, macrophages, eosinophils, mast cells, dendritic cells, and other associated cells identify and elicit protecting responses to invading pathogens through pattern recognition receptors such as toll-like receptors (TLR), nucleotide oligomerization domain (NOD)-like receptors (NLRs), and retinoic acid-inducible gene I (RIG-I)-I-like receptors (RLRs) (Bordignon et al., 2017). Antigen-presenting cells (APCs) such as Langerhans cells (LC), macrophages, and dendritic cells play an essential role in connecting the innate immune response to the adaptive immune system (Woodworth, 2002). LCs are the only APCs that can access the HPV proteins in the epithelial cells of the surface layer (Doorbar et al., 2012). HPV transfection of DCs leads to changes in DC migratory pattern and induces cytokine production, which may suppress immune response to viral antigens (Khaiboullina et al., 2013). Macrophages contribute to the clearance of HPV infection through the production of tumor necrosis factor-alpha and nitric oxide-dependent mechanisms (Routes et al., 2005; Bergot et al., 2011). Natural killer cells have been associated with viral clearance, elimination of HPV infected cells, and cancer prevention in HPV-related carcinogenesis (de Freitas et al., 2017). Although less is known about neutrophils and cervical cancer, recent data demonstrates that high concentrations of neutrophils are strongly associated with poor prognosis and disease progression in women with cervical cancer (Alvarez et al., 2017).

### Adaptive Immune Response

The adaptive immune response is another protective line of host defense against pathogens. It is differentiated into two pathways, the T-helper (Th)1 cell responses that promote cell-mediated immunity and Th2 cell responses that promote humoral immunity (Stanley, 2006). During HPV infection, the antigen is taken up by DCs through phagocytosis and migrates to site lymphoid tissues to activate adaptive immunity *via* the expression of inflammatory cytokines such as IL-1α, IL-1β, IL-6, TNF-α, and IL-12 (Sasagawa et al., 2012). T lymphocytes (CD4+) recognized HPV antigen presented by mature APCs (**Figure 1B**) and undergo proliferation/differentiation (**Figure 1C**), and digest the antigen into shorter peptides or differentiate into effector T cells that interact with naive B cells and produce cytokines to assist in the maturation of B cell responses (**Figure 1D**) (Steele et al., 2002). An anti-viral immune response needs a Th1 cell response which secrete cytokines such as IL-2, IL-12, and IFN-γ to activate immune cells, including the naïve CD8+ T-lymphocytes (**Figure 1E**) that later differentiate into cytotoxic T lymphocytes (CTL) and become effector T-cells that can kill CIN or HPV infected cells (**Figure 1F**) (Sasagawa et al., 2012). CTLs, CD4+ T cells and other Th1 responses have been associated with effective clearance of HPV 16 and HPV 18 (Torcia, 2019), while the lack of these T lymphocyte lineages has been associated with persistent HPV infection and the development of high-grade disease (Pao et al., 1995). Furthermore, Th2 cells produce cytokines such as IL-4, IL-6, IL-8 and IL-10 that help B cells differentiate into plasma cells that produce HPV-specific antibodies into circulation (Woodworth, 2002). Some naive B and T cells transform into HPV-specific memory B and T cells that migrate to the bone marrow to survive as long-lived memory cells and differentiate into plasma cells or activated T cells upon reinfection (Stern et al., 2000). In contrast, previous studies demonstrated that women with high grade HPV+ cervical lesions had an increased IL-17-associated (Th17) response known to be pro-tumorigenic in HPV-associated cancers (Walch-Ruckheim et al., 2015). Activation and recruitment of Th17 cells is mediated by stromal tumor-associated fibroblasts and tumor-derived chemokines (CXCR3) (Walch-Ruckheim et al., 2015). Taken together, these findings suggest that not all innate and adaptive immune lineages are capable of migrating to sites of infection to kill HPV-infected cells, some actually promote progression of cervical lesions to invasive cervical cancer.

## Evasion of Host Immune Responses by HPV

### Innate Immune Evasion by HPV

HPV can deploy various mechanisms to evade host immune responses to establish persistent infections. One of the mechanisms involves altering APC function, including pathogen recognition receptors. High-risk HPV has been suggested to inhibit keratinocyte CCL20 expression, which reduces the ability of LCs to induce a cytotoxic immune response by compromising LCs infiltration (Chandra et al., 2016). In addition, HPV infection has been shown to affect the differentiation of monocytes into mature DCs, leading to altered functionality of DCs (Song et al., 2015). Another evasion mechanism of HPV infection is by inhibiting the recruitment of macrophages and other immune cells into the site of infection by the HPV E6 and E7 proteins. (HPV16 only) (Stern et al., 2000). HPV16 E6 and E7 proteins have been shown to prevent translocation of macrophages *via* downregulation of cytokines (TNF-α and MIP-3α) responsible for activation of these multifunctional cells (Hacke et al., 2010; Bashaw et al., 2017). Hasan *et al.* (2007) demonstrated that HPV-16 downregulated the expression and function of TLR-9 in human epithelial cells (Hasan et al., 2007). These findings suggest that overexpression of oncoprotein may inhibit the dendritic cell function in the epithelium.

Natural killer cells are another key component of the innate immune response that HPV evades. E5 protein has been shown to inhibit CD1d on the surfaces of cells infected with HPV6 or 16, resulting in decreased cytotoxic activity of NK cells. Furthermore, high-risk-HPV genotype (HPV-18) has been associated with suppression of the cyclic guanosine monophosphate-adenosine monophosphate synthase, which plays a role in activating type I interferon genes and production of inflammatory cytokine response (Lo Cigno et al., 2020). The interferons play a role in inhibiting viral replication in the host cells and activating immune cells that can eradicate the infected cells (Doorbar et al., 2015). Studies have demonstrated that E6, HPV16 and 18 evade innate immune response by downregulating the transcription of type-1 IFNs and IFN Kappa secretion (Koromilas et al., 2001; Woodby et al., 2018). More studies on the interaction between HPV proteins and type I IFN response remain critical.

### Evasion of Adaptive Immune Response by HPV

The virus is also able to escape adaptive immune responses. Although the mechanisms by which HPV can evade the host immune system remain unclear, HPV proteins and regulatory cytokines have been suggested as contributors (Westrich et al., 2017). HPV16 E5 and E7 have been reported to reduce expression of interferons and HLA class-1 molecules, resulting in a lack of CTL response against HPV (Stern et al., 2000). In addition, studies have reported high concentrations of an anti-inflammatory cytokine (IL-10) in women with persistent HPV infection compared to those who cleared the HPV infection. Similarly, increased levels of immunosuppressive cells such as transforming growth factor-beta (TGF-β)-producing Tregs have been observed in cervical tissues and vulval intraepithelial neoplasia (VIN) lesions. The increased concentrations of IL-10 and TGF-β in tissues may indirectly undermine T cell function by limiting the ability of APCs to promote CD4^+^ T cell differentiation and proliferation, thereby modulating the adaptive immune responses (Sasagawa et al., 2012). Another mechanism by which CD4+ and CD8+ T cell function is inhibited involves downregulation of surface MHC I expression and the impairment of APC trafficking and maturation (Hilders et al., 1995; Keating et al., 1995; Bontkes et al., 1998). Furthermore, dysregulated expression of IL-8 receptor by HPV16 E6 protein affected the CD8+ mediated response (Cho et al., 2001). Collectively, these finding suggest that APC and T cell response downregulated by immunosuppressive immune response may result in HPV-immune evasion, and consequently lead to persistent HPV infection and the development of high-grade disease

## Association Between Genital Inflammation and HPV

Inflammation is defined as the natural immune response following injury or infection (Ferrero-Miliani et al., 2007; Medzhitov, 2010). It is generally characterised by elevated levels of cellular markers and pro-inflammatory cytokines in the genital tract (Passmore et al., 2016). Host defense mechanisms, including immune mediators in the female genital tract microenvironment, play a role in the clearance and persistence of HPV and the risk of developing cervical cancer (Stanley, 2010; Daud et al., 2011; Łaniewski et al., 2018; Liebenberg et al., 2019; Onywera et al., 2019; Moscicki A-B et al., 2020).

Contradicting data have been reported between HPV and altered cytokine milieu profiles (Łaniewski et al., 2018; Liebenberg et al., 2019, 12). Łaniewski *et al.* (2018) showed a strong association between invasive cervical carcinoma and elevated concentrations of TNF-α, TNF-β, MIP-1α, GMCSF, and IL-10 (Łaniewski et al., 2018). In addition, HPV persistence was associated with chemokine MIP-1α and growth factor GM-CSF that play a significant role in activation and recruitment granulocytes (Marks et al., 2011). Another study showed that women with elevated concentrations of mucosal cytokine interleukin (IL)−10, IL−12, macrophage inflammatory protein (MIP)−1α and TNF−α were less likely to clear any HPV type while low levels of these cytokines (including IL-8) correlated with HPV clearance (12). A positive correlation between elevated concentrations of several cytokines (IL-36γ, MIP-1β, RANTES, IP-10, IL-2, IL-4, Flt-3L, sCD40L) and invasive cervical cancer carcinoma in women with BV were reported (Łaniewski et al., 2018). The β-chemokines (RANTES and MIP-1β), IL-8, IP-10, known to promote immune cell activation and recruitment and have shown to limit cellular entry of pathogens (Mhatre et al., 2012). Increased mucosal cytokine profiles in the reproductive tract of women infected with HPV was also associated with HPV prevalence, clearance, acquisition, persistence and increased HIV acquisition risk (Liebenberg et al., 2019). The apparent association between HPV infection and genital cytokine responses may likely indicate the role of cellular immunity to control HPV infection. Moscicki *et al.* (2020) found that 9 of the 13 cytokines (IL-4, IL-5, IL-10, IL-12, IL-13, IFN-γ, IFN-2α, MIP-1α, and TNF-1α) tested were elevated after the clearance of HPV infection compared to prior visits (Moscicki AB et al., 2020). Production of anti-inflammatory cytokine IL-4, IL-5, IL-10, and IL-13 are known to promote macrophage activation and promote the differentiation of B lymphocytes that are crucial for the production and secretion of antibodies (Fernandes et al., 2015). In contrast, two studies did not show the relation between increased concentrations of pro inflammatory cytokines and HPV acquisition or clearance (Kriek et al., 2016; Shannon et al., 2017). Understandably, while there are no structural conformations on host cells during HPV invasion, HPV also maintains the anti-inflammatory state likely by avoiding the host immunity through disruption of the interplay between infected cells and effector cells (98). There is a need to better understand the cellular or other factors associated with the cytokines in the different HPV status categories.

## Association Between the Vaginal Microbiome and HPV Infection

The vagina and ectocervix are dominated by lactic-acid producing bacteria and cervicovaginal fluids that act as a lubricant that traps invading pathogens (Lee et al., 2013; Aldunate et al., 2015). A genital environment dominated by *Lactobacillus* spp. has been associated with optimal pregnancy outcomes, lack of abnormal vaginal symptoms and urogenital disease, and reduced risk for several STIs, including HPV and HIV (Petrova et al., 2013). In contrast, the opposite is observed in the genital tract dominated by non-optimal vaginal microbiota. Several *Lactobacillus* spp. have been described in the non-BV state, although the most frequent and abundant are *L. crispatus, L. gasseri*,and *L. jensenii (* Ravel et al., 2011; Bik et al., 2019 *)*,. *L. gasseri* enriched microbiome was also associated with rapid rates of HPV clearance (Brotman, 2011). A recent study showed that women with *L.crispatus* enriched microbiome were less likely to have prevalent high-risk HPV infection than women with overgrowth of pathogenic microorganisms such as those linked with BV (*Gardnerella, Atopobium*, and *Prevotella*) (105). Furthermore, *L.crispatus* abundance in the genital tract was also associated with HPV clearance, suggesting the relative association between clearance and *L. crispatus* (105).

BV is a condition characterized by a shift in vaginal microbiota from *Lactobacillus* dominant towards more diverse bacteria, including strict and facultative anaerobes such as *Gardnerella, Prevotella*, and *Sneathia*, often resulting in vaginitis and discharge (105, Cohen et al., 2012). BV has been associated with elevated levels of cytokines (IL-1α, IL-1β, IL-18, IL-7, MIP-1α, MIP-1β, IL-8, MIF, TNF-α, and TNF-β) and cellular (CCR5+CD4+ T cells) biomarkers of inflammation associated with increased HIV acquisition risk (Cohen et al., 2012; Eade et al., 2012). Furthermore, numerous studies have suggested a link between BV and other STIs such as *Chlamydia trachomatis, Neisseria gonorrhea*, and cervical HPV (Cherpes et al., 2003; Brotman, 2011). The ulcerative and highly inflammatory sequelae caused by STIs and/or BV (inflammation) provide biologically plausible mechanisms supporting a possible increased susceptibility to HPV among co-infected individuals. A high relative abundance of *Gardnerella* and *Atopobium vaginae* were associated with CIN (Godoy-Vitorino et al., 2018; Berggrund et al., 2020). Increased abundance of *Sneathia*, *Atopobium*, and *Gardnerella* is associated with incident high-risk HPV infection (Onywera et al., 2021). A study by Lee *et al.* (2013) showed that HPV-infected women had lower *Lactobacillus* species and increased *Fusobacteria* and *Sneathia* compared to HPV-uninfected women (Lee et al., 2013). In agreement with these findings, longitudinal analysis from 32 sexually active women showed that a low *Lactobacillus* community with high proportions of the genera *Atopobium* species were associated with the low rate of HPV clearance (Kyrgiou et al., 2017). While existing evidence suggests the association between HPV infection and genital dysbiosis, justifiable concerns that positive associations merely reflect residual confounding by unmeasured sexual risk behaviors (such as engaging in condomless sex and having sex with uncircumcised partner) still exists (Averbach et al., 2010). Thus, further research investigating this interplay is warranted. Detailed understanding of the genital microbial composition and structure in women with HPV infection may help identify the causal connections between microbiota, HPV infection, and cervical cancer.

## Association Between HPV and HIV

### HPV Infection and HIV Risk

Epidemiological and meta-analyses data suggest that sexually transmitted infections like herpes, gonorrhoea, chlamydia, and syphilis can increase the risk of HIV acquisition (Fleming and Wasserheit, 1999; Ward and Rönn, 2010). Other biological correlates of HIV risk that have been described to date is mucosal pro-inflammatory cytokines and BV-associated bacteria that, if altered prior to infection, were associated with higher risk of acquiring HIV (Anahtar et al., 2015; Masson et al., 2015; Yegorov et al., 2019). However, less is known about the mechanism by which HPV infection might increase HIV acquisition risk.

In addition to its role as a biological factor in the development of anogenital cancers, HPV may also be an important co-factor in the increased risk of HIV acquisition in women. Overall, HIV infection risk doubled in women with prevalent HPV infection, with either oncogenic or non-oncogenic HPV genotypes (Liu et al., 2018). Another study showed a significant association between high-risk HPV genotypes and HIV acquisition (Looker et al., 2018). Persistent HPV infection has been associated with an increased biomarkers of HIV acquisition, and the causal link is still not well-understood (Chikandiwa et al., 2017). Higher frequency of the CD4+ T cells in the stroma and epithelium are closely associated with HPV lesion regression (Monnier-Benoit et al., 2006) and consequently increasing mucosal HIV target cell frequency and activation (McKinnon et al., 2011). In addition, pro-inflammatory cytokines essential for HPV clearance are also known to increased risk of HIV acquisition (Masson et al., 2015; Liebenberg et al., 2019). Further research is still needed to explore the association between HPV and HIV and validate HPV as a potential risk factor for HIV acquisition, and if found to be true, these may highlight the importance of decreasing HPV burden in settings with high prevalence to curb HIV infections.

### HPV Infection in Women Living With HIV

HIV infection has also been associated with the development of CIN2, CIN3, and invasive cervical carcinoma in HPV infected population (Clifford et al., 2017). Although multiple types of HPV have been associated with HIV infection, HPV16 is the commonest cause of cervical carcinoma in HIV infected population (Dreyer, 2018). Studies have reported that women living with HIV (WLWH) are more likely to be infected with high-risk HPV and multiple HPV genotypes, resulting in the development of pre-invasive lesions that, if left untreated, can develop into invasive cervical cancer (98, Liu et al., 2018). WLWH have up to five times more cervical cancer than HIV-uninfected (Liu et al., 2018). In two large prospective studies that assessed the prevalence of HPV genotypes in women living in the United States, HPV 6 or 11 was 3.6 and 5.6 times high in WLWH compared to their HIV-uninfected counterparts, respectively (Bruni et al., 2010; Bogale et al., 2020). Furthermore, patients living with HIV were at increased risk of cervical abnormalities and onward HPV transmission due to high prevalence of high HPV viral load (Looker et al., 2018). Given the growing evidence of an increased risk of cervical cancer in WLWH, regular HPV screening and possibly treatment for cervical cancer is needed to effectively control HPV and its adverse sequelae.

## Conclusion

While there is substantial progress in increasing vaccine access and immunization coverage, young women, particularly in sub-Saharan African remain disproportionately infected by HPV. Factors that may render women more vulnerable to HPV infection have not been fully characterized. This review showed that the vaginal microbiome, cellular, and cytokine markers of inflammation are some of the biological markers that are associated with neoplastic disease in cervical carcinogenesis. While significant progress has been made in understanding how HPV evades immunity, mechanistic studies on how risk factors influence host-mucosal microenvironment and viral persistence are warranted. The role of other biological risk factors such as intravaginal insertion practices (for hygenic and sexual enhancement purposes) are less studied and could be important drivers of HPV risk in young women. There remains a need to conduct more preclinical models to understand how biological risk factors might block efficient HPV clearance from the mucosa and pave the way for cervical cancer.

## Author Contributions

All authors contributed to the article and approved the submitted version.

## Funding

This review was conducted as part of the DST-NRF Centre of Excellence (CoE) in HIV Prevention, supported by the Department of Science and Innovation and the National Research Foundation (grant 96354). LN was funded by DST-NRF CoE in HIV Prevention (grant 96354) and Poliomyelitis Research Foundation Research Bursary (bursary# 21/73). SN was supported by Columbia University-Southern African Fogarty AITRP Programme (grant# D43TW00231), National Research Fund Thuthuka Research Grant (grant# TTK160510164586), and Poliomyelitis Research Foundation Research Grant (grant# 16/17). GM was funded by Poliomyelitis Research Foundation Research Grant (grant# 18/16) and National Research Foundation Grant (grant# SFP180507326699). AM was supported by National Research Foundation Grant (grant# PDG210309589501)

## Conflict of Interest

The authors declare that the research was conducted in the absence of any commercial or financial relationships that could be construed as a potential conflict of interest.

## Publisher’s Note

All claims expressed in this article are solely those of the authors and do not necessarily represent those of their affiliated organizations, or those of the publisher, the editors and the reviewers. Any product that may be evaluated in this article, or claim that may be made by its manufacturer, is not guaranteed or endorsed by the publisher.
